# Investigating Fungal Outbreaks in the 21st Century

**DOI:** 10.1371/journal.ppat.1004804

**Published:** 2015-05-21

**Authors:** Anastasia P. Litvintseva, Mary E. Brandt, Rajal K. Mody, Shawn R. Lockhart

**Affiliations:** Mycotic Diseases Branch, National Center for Emerging and Zoonotic Infectious Diseases, Centers for Disease Control and Prevention, Atlanta, Georgia, United States of America; Duke University Medical Center, UNITED STATES

## What Causes Fungal Outbreaks?

Public attention has been drawn to recent high-profile outbreaks of mycotic diseases, such as those of fungal meningitis and other infections linked to contaminated steroids [1] and an outbreak of necrotizing cutaneous mucormycosis linked to a tornado [2]. However, fungal outbreaks are more common than most people appreciate, and reports of outbreaks caused by unusual fungal pathogens are increasing. The Mycotic Diseases Branch at the Centers for Disease Control and Prevention (CDC) investigates 3–6 fungal outbreaks per year, many of which are caused by rare fungi with limited diagnostic and treatment options. This is a considerable increase from the 1990s, when the Branch investigated 1–2 hospital-based outbreaks per year, generally caused by yeast and traced to a single source. Although the exact reasons for this increase are unknown, the increased number of patients with impaired immune system may have contributed to this trend.

The majority of fungal outbreaks can be attributed to either environmental exposure or a contaminated product (Table 1). For example, two recent outbreaks were linked to contaminated medications. In 2012, two medications produced by a single compounding pharmacy in Florida were contaminated with *Fusarium* sp. and *Bipolaris* sp., respectively, shipped to 15 states, and injected into the eyes of patients undergoing vitrectomies. As a result, 47 patients developed endophthalmitis, and most lost vision [3]. In the 2012 fungal meningitis outbreak, methylprednisolone acetate (MPA) contaminated with *Exserohilum rostratum* and several other microorganisms was shipped to 23 states, potentially exposing nearly 14,000 individuals to this contaminated medication. As a result, 752 people developed meningitis, arachnoiditis, or spinal/paraspinal abscesses, and 64 patients died, making this the deadliest fungal outbreak to date [1].

**Table 1:** Examples of fungal outbreaks.

Infection	Agent	Year	Number of People Infected (Died)	Source	Genotyping Method	Reference
Keratitis	*Fusarium* sp.	2005	45	unknown	MLST	[11]
Cutaneous Mucormycosis	*Rhizopus delemar*	2008–2009	5(5)	hospital linens		[4]
Coccidioidomycosis	*Coccidioides immitis*	2009	3	infected organ donor	WGST	[14]
Necrotizing Cutaneous Mucormycosis	*Apophysomyces trapeziformis*	2011	13(5)	tornado debris	WGST	[2,5]
Fungemia and Other Infections	*Saprochaete clavata*	2011–2012	30(22)	unknown	WGST	[17]
Endophthalmitis	*Fusarium incarnatum-equiseti*, *Bipolaris hawaiiensis*	2012	47	Brilliant Blue G and triamcinolone	MLST	[3]
Meningitis and Other Infections	*Exserohilum rostratum*	2012–2014	752(64)	MPA	WGST	[1,15]
Surgical Site Infections	*Bipolaris spicifera*	2013	21	environmental exposure	MLST	
Fungemia	*Cryptococcus neoformans*	2013	5(3)	unknown	MLST	

Environmental exposure is the other common cause of fungal outbreaks [2,4]. For example, a recent cluster of *Rhizopus delemar* infections in a children’s hospital in New Orleans was linked to contaminated linens [4]. In this outbreak, five children died from cutaneous mucormycosis, and *R*. *delemar* was isolated from linens, linen shelves, and bins at the hospital [4]. In another example, 13 people developed necrotizing cutaneous mucormycosis caused by *Apophysomyces trapeziformis* after receiving puncture wounds caused by flying debris during a tornado [2]. Although *A*. *trapeziformis* was not recovered from the local environment, whole genome sequence typing (WGST) of the isolates showed that at least three different strains were involved, suggesting environmental exposure [2,5]. In addition, many other outbreaks and clusters caused by dimorphic and other fungal pathogens have been linked to environmental exposure [6–8].

## What Role Does Epidemiology Play in Investigating Fungal Outbreaks?

**Fig 1:**
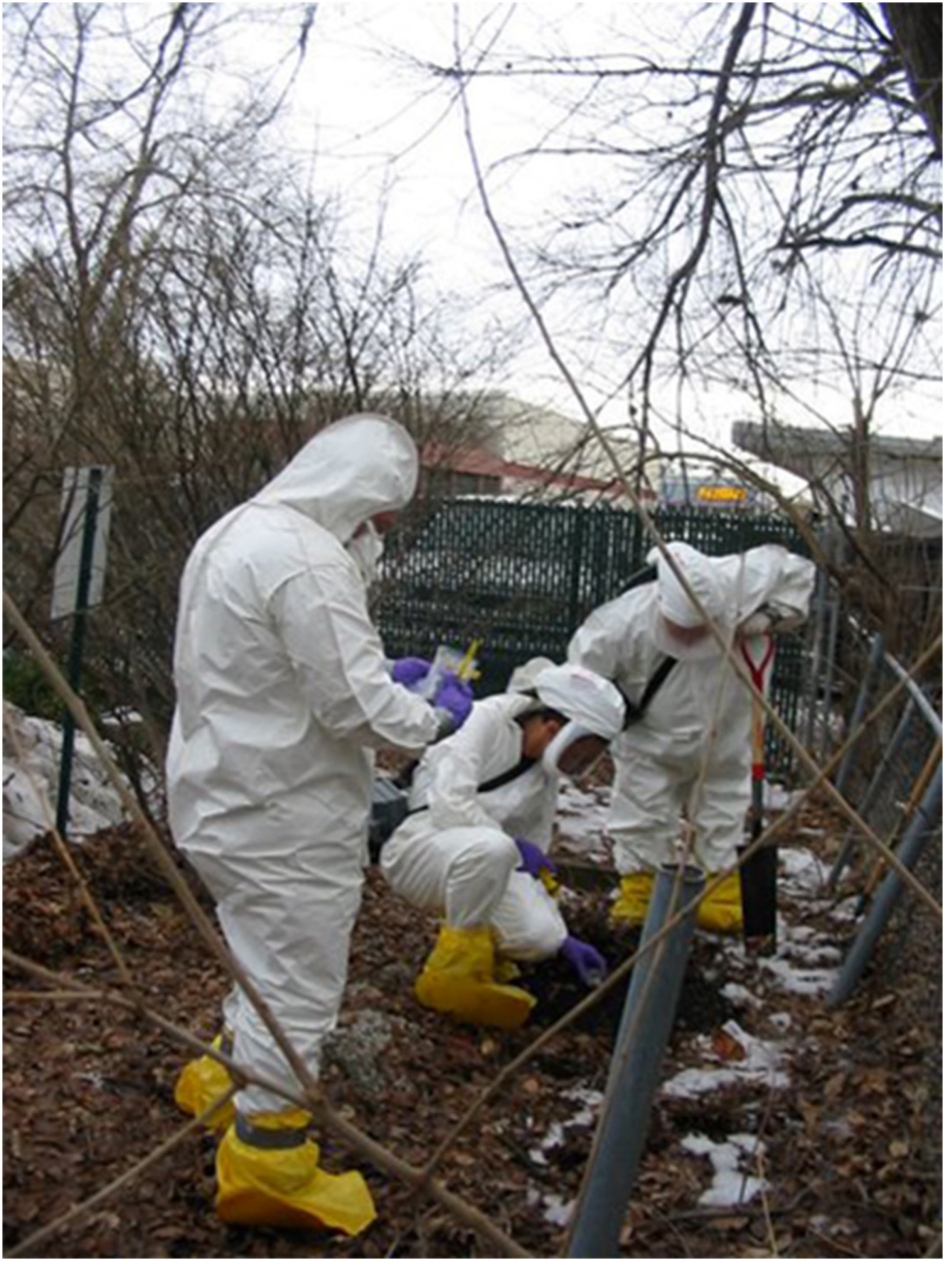
CDC epidemiologists are collecting environmental samples in a histoplasmosis outbreak investigation.

Descriptive epidemiology (i.e., detailed assessment of patients’ demographic characteristics, clinical histories, and the geographic and temporal distribution of cases) is essential for generating hypotheses about the potential source of infection. For example, cases occurring over several weeks to months and scattered geographically suggest a common source outbreak that involves a widely distributed product, especially when case patients have undergone similar medical treatments [1,3]. Conversely, cases occurring among persons with exposure to a common location suggest environmental transmission. Specifically, environmental transmission may be likely among patients with invasive mold infections cared for in the same hospital [4,9,10] or among people with dimorphic fungal infections who participated together in outdoor activities [6,8]. Hypotheses generated through descriptive epidemiology can be tested through analytical epidemiological studies and by microbiological testing of suspected sources (Fig 1).

## Why Is Molecular Genotyping Important?

Molecular genotyping complements epidemiological findings. Whereas at least some isolates from common source outbreaks are expected to be genetically identical [1,3], environmental transmission is often associated with multiple strains or species [5,11]. Thus, molecular genotyping is a powerful tool to support or refute epidemiologically generated hypotheses. For example, results of molecular typing directly influenced the response to a cluster of *Bipolaris* sp. infections detected among patients recovering from cardiothoracic surgeries in three hospitals in Texas, Arkansas, and Florida (United States) that was recently investigated by the CDC. Because of the similarity of the patient population, a common product was suspected but could not be identified. However, multilocus sequence typing (MLST) revealed that none of the patients shared strains with the same genotype and that at least two species were involved, strongly supporting environmental exposure rather than a common source. These molecular results provided critical evidence during an early stage of the investigation and focused investigators on the environment rather than on medications and devices used by these patients.

Similar results were obtained when molecular typing was used to investigate a multistate outbreak of *Fusarium* keratitis, in which a case-control study implicated a specific type of contact lens solution and suggested a common source outbreak, although *Fusarium* was not isolated from intact product or the production facility. However, the identification of 19 distinct genotypes of *Fusarium* from case patients provided evidence for independent contamination events in case patients’ local environments [11].

## How Can WGST Help?

When available, conventional methods of molecular genotyping, such as MLST, can rapidly generate results; however, identical patterns obtained by these methods are often difficult to interpret, because they can be attributed to both common origin as well as to the low discriminatory power of the typing method. In particular, conventional genotyping methods are often unable to differentiate among strains from clonal populations with low genetic diversity, such as *Cryptococcus neoformans* or *Cryptococcus gattii* [12,13]. For example, in a recent cluster of *C*. *neoformans* infections in an Arkansas hospital investigated by the CDC, three of five cases of cryptococcosis were caused by isolates with identical ST5 (A5/M5) genotypes, which is also one of the most common MLST genotypes among environmental and clinical strains [13], making it impossible to determine whether these strains were likely to have been acquired independently or from a common source. In addition, certain genotyping methods, such as microsatellite typing, may generate homoplasy, patterns that look indistinguishable but are not related by descent. Furthermore, for the majority of fungi, conventional molecular genotyping methods are simply not available.

WGST provides a highly sensitive tool for molecular genotyping that can be applied to any pathogen without prior knowledge of the genome, which is especially useful for investigating outbreaks caused by rare fungal pathogens for which population structure information may be unavailable. This method has recently been applied to investigate several fungal outbreaks: (i) to confirm molecular identity of *Coccidioides immitis* from three organ recipients who shared the same donor [14], (ii) to investigate genetic relationships among isolates of *E*. *rostratum* from patients and contaminated methylprednisolone [15], (iii) to confirm genetic identity between isolates of *C*. *immitis* from soil in Washington state and a case patient with coccidioidomycosis acquired in that state [16], (iv) to demonstrate multiple origins of the rare mold *A*. *trapeziformis* in the tornado-associated cluster of necrotizing cutaneous mucormycosis [5], and (v) to demonstrate genetic identity among strains of *Saprochaete clavata*, a highly unusual fungal pathogen, associated with a multicenter outbreak in France [17].

The discriminatory power of WGST allows estimation of genetic relatedness among strains of pathogens without prior knowledge of the underlying population structure. For example, WGST analysis of *E*. *rostratum* isolates from the fungal meningitis outbreak indicated that 19 isolates from patients and contaminated medication lots had identical genomes of 33.8 Mb and no more than two single nucleotide polymorphisms (SNPs) differentiated any two isolates, confirming a likely single origin of the outbreak strains. By contrast, more than 20,000 SNPs were detected between any two control strains of *E*. *rostratum*, confirming the genetic diversity among unrelated strains [1,15]. Conversely, genetically identical isolates of *A*. *trapeziformis* as well as isolates separated by thousands of SNPs were identified when WGST was used to investigate the etiology of necrotizing cutaneous mucormycosis, which was consistent with environmentally acquired infections [2,5].

## What Are the Limitations of WGST and What Is the Future of Fungal Outbreak Investigations?

Although the results of WGST can significantly enhance epidemiological investigations, this method is still unacceptably slow for most real-time investigations. For example, WGST results for the *Exserohilum* outbreak were generated 6 months after the initial investigation was completed and therefore provided mostly confirmatory data [15]. In order for WGST to become applicable for real-time investigations, time and cost of generating and analyzing WGST data need to be reduced. WGST methods are already widely used for investigating bacterial and viral outbreaks. However, most fungal genomes are at least ten times larger than those of bacteria and viruses; therefore, considerably more time and resources are needed to generate and process fungal genomes.

Development of a curated public database containing assembled genomes of major fungal pathogens will significantly accelerate analyses and implementation of WGST into public health by providing reference genomes and control strains for assessing genetic diversity in a population as well as facilitate data sharing among institutions. It is also possible that for some fungi, WGST can be substituted with a high-density MLST or a targeted SNP-based typing system that can provide as much information as WGST. As population genomic data for fungal pathogens accumulate, targeted SNPs, MLST loci, or both can be selected for high-resolution targeted genotyping for population genetic studies and molecular epidemiological investigations.

The other significant limitation of current WGST is the difficulty in detecting and identifying fungal DNA directly in human clinical samples against the human DNA background. Proteomic and metagenomic methods have been used for culture-independent detection and typing of viral and bacterial pathogens [18–20], and these methods are being adapted for fungi [21]. Accumulation of fungal genomic data and the development of a WGST database for fungal pathogens will provide a necessary framework for developing metagenomic tools for detection and typing of fungi in clinical samples. In addition, genomic data will facilitate basic research aimed at understanding pathogenesis and improving antifungal therapies. As vulnerable patient populations increase and exposure to pathogenic fungi continues, the number of fungal outbreaks is also likely to increase. We anticipate that novel molecular tools coupled with thorough epidemiological investigation will continue to assume greater importance in recognizing, stopping, understanding, and preventing fungal outbreaks in the future.
